# Implantable loop recorders can detect paroxysmal atrial fibrillation in Standardbred racehorses with intermittent poor performance

**DOI:** 10.1111/evj.13372

**Published:** 2020-11-23

**Authors:** Rikke Buhl, Sarah D. Nissen, Marie L. K. Winther, Sofie K. Poulsen, Charlotte Hopster‐Iversen, Thomas Jespersen, Prashanthan Sanders, Helena Carstensen, Eva M. Hesselkilde

**Affiliations:** ^1^ Department of Veterinary Clinical Sciences Faculty of Health and Medical Sciences University of Copenhagen Taastrup Denmark; ^2^ Department of Biomedical Sciences Faculty of Health and Medical Sciences University of Copenhagen Copenhagen Denmark; ^3^ Centre for Heart Rhythm Disorders Royal Adelaide Hospital and University of Adelaide Adelaide Australia

**Keywords:** cardiac arrhythmia, cardiology, horse, implantable loop recorder, paroxysmal atrial fibrillation

## Abstract

**Background:**

Limited information is available on paroxysmal atrial fibrillation (PAF) in the horse. Indeed, undiagnosed PAF could result in poor performance. Due to the intermittent occurrence, PAF is difficult to diagnose. However, implanting a small ECG device (implantable loop recorder, ILR) subcutaneously, allows the continuous and automatic detection of PAF.

**Objectives:**

The aim was to investigate the potential of ILRs as a tool for diagnosing PAF in horses with poor performance.

**Study design:**

Prospective field study.

**Methods:**

Twelve racing Standardbred trotters with intermittent reduced performance (mean age: six years) were enrolled prospectively. The ILR was implanted subcutaneously at the fifth or sixth left intercostal space and data from the ILR was collected during the study period in which the horses were followed for a median duration of 7.5 month (range 6‐28).

**Results:**

The ILR was able to detect PAF in four out of twelve racehorses. The ILR also detected sustained atrial fibrillation (AF) in one horse during the study. The ILRs rely on RR detection and R waves were correctly identified in 96%. One hundred episodes were categorised as AF by the ILRs and subsequently visual ECG inspection categorised 12 as sinus rhythm (SR), 28 as sinus arrhythmia (SA), 14 as other arrhythmias and 46 as AF episodes. The Root Mean Square of the Successive Differences (RMSSD) values were significantly increased for AF compared to SR and SA.

**Main limitations:**

Few horses included and duration of study period varied among the horses. Further it was not possible to assess the sensitivity of the device in the current study and the ILRs proved to have a high rate of false positive misclassifications.

**Conclusions:**

This study indicates that ILRs can be used for detection of PAF episodes and could be a useful ECG tool for horses presenting with poor performance. This methodology provides a platform to facilitate the long‐term assessment of AF development and quantification of AF burden in horses. Further studies including both healthy and poor performing horses are needed in order to learn more about PAF prevalence in racehorses.

## 1 INTRODUCTION

Atrial fibrillation (AF) is detrimental to the performance in athletic horses exercising at maximal capacity^1^ and is the most common pathological arrhythmia related to poor performance.^2^

The diagnosis of AF is based on an irregularly irregular rhythm detected on cardiac auscultation which is confirmed by electrocardiography (ECG) revealing irregularly irregular RR intervals, absence of P waves and the presence of f waves. The RR interval differences reflecting the beat‐to‐beat variance can be calculated by the Root Mean Square of the Successive Difference (RMSSD) and thereby quantify the irregularity of the ventricular beats and differentiate sinus rhythm (SR) from AF, which graphically can be shown by either a Poincaré or Lorenz plot.^3^

Atrial fibrillation can be paroxysmal (PAF) where the horses spontaneously cardiovert to SR within 48 hours.^2^ Due to the short‐lasting and unpredictable nature of PAF it is difficult to diagnose as it would require long‐term Holter ECG recordings. In the past, efforts have been made to overcome this and recently, a new computerised complexity analysis of apparently normal SR ECGs were able to identify PAF horses.^4^ Also, handhold ECG devices are now available, making direct detection possible.^5^ Early recognition of AF is essential as long lasting AF becomes more resistant to treatment.^6^ In people, AF is generally considered to be a progressive condition, initially occurring in a paroxysmal form, then as persistent AF and finally as permanent AF where cardioversion is not possible.^7^ Studies have shown that AF progresses in a similar way in horses. ^8^ However, only few cases of PAF in horses have been described^9^, ^10^, ^11^ and therefore the prevalence or the potential progression from PAF to persistent AF is unknown.

Recently, we described that implantable loop recorders (ILRs) can detect induced AF in horses.^12^ The ILR continuously obtains an ECG and through dedicated algorithms the ILR automatically records predefined arrhythmic episodes based on changes in the RR interval.[13; 14] Unlike Holter ECG recordings, no continuous ECG is provided, but a report on detected arrhythmias including a two minute ECG example for each episode is stored. In humans, ILRs are used to detect PAF or AF recurrence in asymptomatic patients, with greater diagnostic yield than conventional Holter ECG.^13^, ^14^, ^15^, ^16^, ^17^

The aim of the study was to investigate the potential of ILRs as a tool for diagnosing PAF in horses with poor performance by visual evaluation of the stored ECGs, the heart rate viability (HRV) parameter RMSSD and Poincaré plot.

## 2 MATERIALS AND METHODS

### 2.1 Horse population and cardiac examination

Twelve race‐fit Standardbred trotters, 11 geldings and one mare (mean age 6 years [range 3 ‐ 8 years]) were recruited from race trainers in Denmark in a prospective field study. The only criteria for inclusion of a horse was intermittent poor performance during racing or training of unknown reason. One horse (#02) had been electrically cardioverted from persistent AF to SR 24 months prior to inclusion and was back in full race training at the time of enrolment. All horses underwent cardiac evaluation consisting of a clinical examination, standard echocardiography (2‐D, M‐mode and colour flow Doppler with a 1.5 MHz phased array probe, Vivid IQ, GE Healthcare Danmark A/S) where valvular regurgitations, left ventricular and left atrial size were measured as previously described,^18^ a five minute resting ECG (Televet100, KRUTECH Televet, Kruuse A/S) and a field exercise test on the racetrack (warm‐up followed by 1,600 m with heart rate (HR) above 200 bpm) equipped with an ECG device (Televet 100, KRUTECH Televet, Kruuse A/S). The ECG electrodes were placed as previously described.^18^ The echocardiographic examination as well as the resting and exercise ECG were analysed in order to exclude other cardiac diseases as a cause of poor performance. The horses were enrolled from December 2017 to October 2019. Last interrogation was conducted 1 May 2020. The study was approved by the local ethical committee at University of Copenhagen and written owner consent was obtained.

### 2.2 Implantation of the ILRs

**Figure 1:**
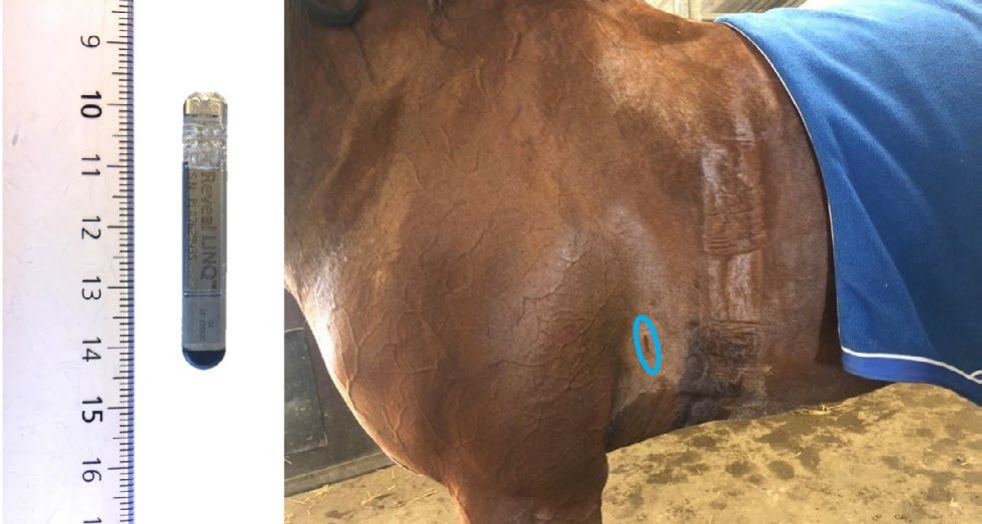
Picture of the implantable loop recorder (ILR) used in the study (Medtronic Reveal LINQ^TM^). The blue circle depicts where the ILR is placed in a horse. Caudal to the ILR the damped area shows where the girth is located, illustrating that the ILR is not in contact with the girth

Following the exercise test, the horses were sedated with detomidine (10 µg/kg, Domosedan^®^vet, Orion Pharma Animal Health A/S) and butorphanol (10 µg/kg, Torbugesic®, Dolorex^®^ vet, MSD Animal Health). The Medtronic Reveal LINQ^TM^ ILRs (Reveal LINQ^TM^, Medtronic Inc.) were implanted subcutaneously in the fifth or sixth left intercostal space at the level of the shoulder joint approximately 15 cm above the level of the left olecranon (Figure 1) as previously described.^12^ The location was adjusted according to the girth position of the individual horse. The ILRs were repurposed but all had battery status “good” at implantation. The horses were treated with 1.1 mg/kg flunixin intravenously (Finadyne^®^vet, MSD Animal Health) after implantation of the ILR, and the horse owner or trainer was instructed to monitor the wound for swelling, exudate and signs of pain during the study period. Following implantation, interrogation with the ILR was performed using the programming device (2090 Medtronic) (Reveal LINQ^TM^, Medtronic Inc.). The ILR was programmed to perform ventricular sensing (VS) using the following predefined settings; Sensing threshold (defines the minimum height of a signal to be detected as a heartbeat [R wave]) = 0.035 mV, and blanking after sense (defines the minimal time before a new R‐wave can be detected) = 150 ms. In this study, we focused on automatic detection of all AF episodes (settings: *AF only*, *AF detection less sensitive* and *nominal ectopy rejection*) and therefore all other arrhythmia diagnoses were turned off. The AF detection algorithm is based on both an RR interval and a P‐wave evidence score. The algorithm conducts a rhythm classification every two minutes. When an episode is detected, the ECG of the first two‐minute period is stored on the device. The device can store up to 14 episodes that includes an ECG example. When the memory is full, the earliest episode is overwritten by newer episodes. Finally, a list of *all* the episodes is stored but only the 14 latest ECGs are available. A detailed description of how the ILR record and store arrhythmia episodes and ECG traces has previously been published.^12^

### 2.3 Data collection

Interrogations with data transfer from the ILR were performed at implantation and during the study period. At each interrogation, a protocol including a list with the last 30 episodes as well as AF burden measured as hours/day was exported as a PDF file onto a memory stick. At each interrogation, the amplitude of the ECG was noted. After the interrogation all the stored data were automatically removed from the ILR so that new episodes could be stored. The owner/trainer was instructed to continue training and racing of the horses during the study period. If a PAF episode was registered at interrogation, the trainer was asked if the horse was exercising as they expected and if they could recall poor performance at that specific time point. Data on racing attendance during the study was collected from the national racing database.

### 2.4 Data analysis

The ECGs were manually analysed by reviewing the ventricular sensing (VS) in the ECGs for misclassifications and to determine whether correct detection of AF had been done. Every RR interval was listed in Microsoft Excel^®^ and if false sensing was made, the true RR interval was manually corrected.

The VS misclassifications were further divided into the following categories: sensing on T wave; sensing on P wave; sensing on f wave; and sensing on artefact. In addition, if no sensing occurred on the R wave, this was categorised as under sensing VS.

All ECGs available were visually inspected and grouped into the following categories: sinus rhythm (SR); sinus arrhythmia (SA); other arrhythmias (OA), (including second‐degree atrio‐ventricular block [2AVB]; atrial or ventricular premature depolarisations [APD, VPD]); and AF. In cases of simultaneous appearance of SA and 2AVB in the same episode this was classified as OA. If marked artefacts were present, this was noted.

The corrected RR intervals recorded from the ECGs were subsequently used to calculate the HRV parameter RMSSD and to create a Poincaré plot as previously described.^3^ In brief, the RR intervals were extracted from the stored 2 minute ECG strips into Microsoft Excel^®^ and the corresponding RMSSD were calculated using the following mathematical formula:

$$RMSSD=\sqrt{\frac{1}{N‐1}\sum_{j=1}^{N‐1}{\left({\mathrm{RR}}_{j+1}‐{\mathrm{RR}}_{j}\right)}^{2}}$$

### 2.5 Data analysis

Statistical analysis was performed using Microsoft Excel^®^ and GraphPad Prism, Version 8 (GraphPad software Inc.). Data were tested for normal distribution by Shapiro‐Wilk test and visual inspection of QQ plots. Parametric data are presented as mean ± standard deviation (SD) whereas nonparametric data are presented as median along with minimum and maximum values. As the purpose of the study was to evaluate if ILRs were able to detect PAF in poor performing horses, each episode registered by the ILR was treated as an independent variable regardless of which horse they were registered from. One‐way ANOVA with Tukey's multiple comparisons test was used to analyse the differences in the RMSSD values for the categories of SR, SA, OA and AF episodes. *P* < .05 was considered statistically significant.

## 3 RESULTS

Fifteen ILRs were inserted in 12 Standardbred horses during the study period. In two horses the ILR was replaced during the study as they ran out of battery. The old ILR was removed, and the new was placed in the existing subcutaneous pocket without any complications. One horse had the ILR removed eight months after implantation at the end of the study, as the ILR mistakenly was implanted in the seventh intercostal space thereby causing interference with the girth. For unknown reasons, one horse lost its ILR device twice, of which the first one was replaced but not the second one. The remaining horses were not affected by the ILRs. The horses were followed for a median duration of 7.5 month (range 6‐28) and they continued their normal training (mean number of races 13 ± 10) during the study. Interrogations with data transfer from the ILR were performed at implantation and a median of five times (range 3‐10 times) during the study period.

### 3.1 Cardiac examination at rest and during exercise

Body temperature, heart rate and respiratory rate were all within normal limits. One horse (#05) was diagnosed with persistent AF at inclusion and the ILR was implanted while the horse was in AF. The horse was subsequently cardioverted to SR 29 days after implantation using quinidine sulphate and the horse continued to be enrolled in the study after cardioversion. One horse had a grade 2/6 systolic murmur with PMI over the mitral valve. The ECGs obtained at inclusion were without comments except for one ECG with multiple episodes of advanced 2AVB, one horse had one APD and finally the horse (#05) diagnosed with AF was identified. All horses reached a HR above 200 beats/min during the exercise test except that the horse with persistent AF was not subjected to an exercise test. One horse had multiple single/couplets VPDs immediately after exercise, two horses had two single VPDs and one horse had two APDs during the exercise test. Echocardiographic examination showed trivial/mild tricuspid (n = 6), mitral (n = 5), pulmonic (n = 1) and aortic regurgitation (n = 2). Values of the left ventricle and left atrial size were within normal limits.^18^

### 3.2 Classification of episodes

**Figure 2:**
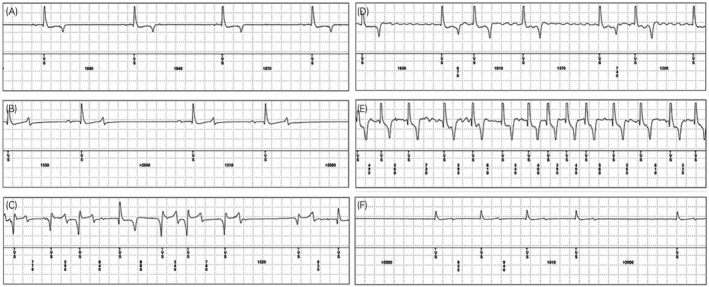
Examples of electrocardiograms (ECGs) recorded by the implantable loop recorder (ILR) in sinus rhythm (A), sinus arrhythmia (B), ventricular premature depolarisations (C), atrial fibrillation (AF) at rest (D), AF at high heart rate after racing (E) and AF recorded at low amplitude (F). Note that the classification of the arrhythmias were based on 2 minute ECG recordings. VS: Ventricular sensing

The ILRs detected a total of 149 episodes as suspected AF. Of the 149 episodes, 49 episodes were overwritten by new episodes and were therefore only stored as summaries without an available ECG. These overwritten episodes all occurred in the horse with persistent AF (#05) before cardioversion and these ECGs could therefore not be assessed manually. The ECGs of the 100 remaining episodes of suspected AF were subsequently visually inspected by two veterinary clinicians and categorised (examples Figure 2, Figure S1 and S2). A complete list of arrhythmias diagnosed in each horse is provided in Table S1.

### 3.3 Paroxysmal atrial fibrillation episodes detected by the ILR

Paroxysmal AF was detected in four out of 12 horses included in the study (Table 1). Despite detecting PAF in these horses they continued their normal training and racing and were not treated with any medication. The ILR saved 29 correctly identified AF episodes from the four horses in total (Horse #01: five episodes, #02:16 episodes, #10: five episodes and #12: three episodes). Visual inspection of the ECGs confirmed AF (Figure 2, Figure S1). Due to the time of occurrence and overall duration of the 29 PAF episodes, these episodes were characterised as eight cases of PAF lasting from eight minutes and up to 11.5 hours. Three of the AF episodes occurred during training and two episodes during racing, whereas the rest occurred during resting periods (Table 1). In all cases where the AF episodes occurred in relation to exercise the owners reported that the horses did not perform well. From the horse (#05) that was in persistent AF at the time of inclusion, 66 AF episodes were registered over the entire study period of which 17 ECGs could be assessed as the remaining 49 ECGs were overwritten. Hence, the ILRs correctly identified a total of 46 AF episodes of the 100 registered, whereas the remaining 54 were falsely detected as AF. Three horses (#03, #11 and #12) had one or more episodes registered as AF that were multiple singlets or couplets of VPDs (Figure 2C and Figure S2). Sinus arrhythmias and OA were identified in seven horses (Horse #01, #02, #03, #05, #10, #11 and #12), (Table S1). Of the 28 episodes identified as SA, 20 of these occurred in the horse (#05) that was medically cardioverted after insertion of the ILR. The SA episodes started 10 days after cardioversion and occurred intermittently over the following 45 days and thereafter no episodes were registered in the following months. Three horses had SR episodes registered as AF (Horse #01, #02 and #03) all of which were recorded at high HR (>60 bpm) and the five of the 12 SR episodes were dominated by artefacts leading to false positive registration of AF.

**Table 1:** Episodes of paroxysmal atrial fibrillation (PAF) in four horses with date, time of atrial fibrillation (AF) onset and AF duration

Horse	Date	Time AF onset	AF duration	Poor performance noted by the trainer at the time of PAF
#01	03.08.2018	05:50	7 h 52 min	Yes ‐ training
	21.12.2018	10:48	9 h 40 min	Yes ‐ training
#02	21.07.2019	07:50	1 h 36 min	Unknown
	15.10.2019	19:43	0 h 8 min	Unknown
	21.11.2019	00:45	7 h 5 min	Unknown
#10	24.03.2020	04:32	4 h 56 min	Yes ‐ training
#12	09.12.2019	19:17	10 h 5 min	Yes ‐ racing
	28.04.2020	16:21	11 h 32 min	Yes ‐ racing

If the trainer had noticed poor performance at that specific date this was registered as well.

### 3.4 Detection of R waves by the ILR

For the ECGs stored by the ILR, the amplitude of the R waves ranged between 0.35‐0.80 mV and the ILR was able to correctly identify R waves (Figure 2) in 9,018 of 9,440 marked R waves (95.5%). In total, 422 beats were falsely marked as R waves and of these 259 (61%) were falsely marked on the T wave, 28 (7%) on the P wave, 45 (11%) on the f wave and finally 90 (21%) on artefacts. In addition, 177 R waves were not identified by the ILR resulting in under sensing of these beats.

**Figure 3:**
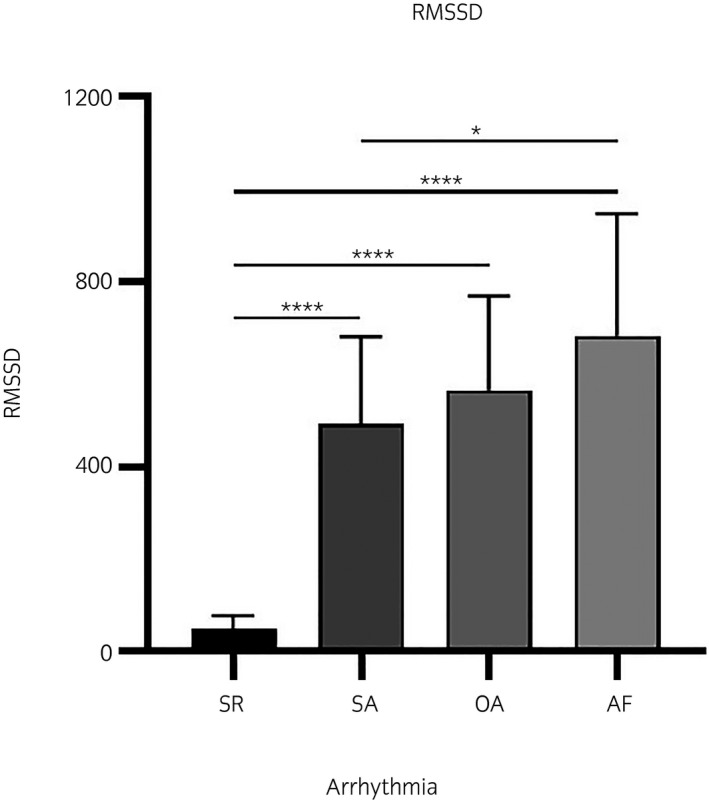
Mean ± SD of the Root Mean Square of the Successive Differences (RMSSD) calculated from two minute episodes recorded in sinus rhythm (SR), sinus arrhythmia (SA), other arrhythmias (OA) and atrial fibrillation (AF). Asterisks (*) indicate significant differences *P < .05, ***P < .0001 and ns = non significant

**Figure 4:**
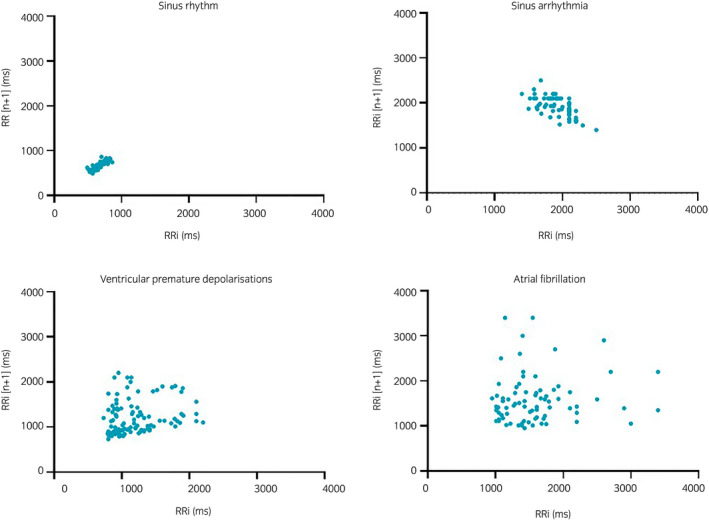
Examples of the heart rate variability in Poincaré plots from sinus rhythm, sinus arrhythmia, ventricular premature depolarisations and atrial fibrillation. RR intervals (RRi) are plotted against their preceding RR interval

The mean RMSSD and duration of the arrhythmia episodes are listed in Table 2. The RMSSD was increased in AF compared to SR, *P* < .0001, and AF compared to SA, *P* = .03. Also RMSSD for OA and SA were increased compared to SR, *P* < .0001. No difference in RMSSD was found between AF and OA, *P* > .05 (Figure 3). The RR distribution is illustrated by the Poincaré plots (Figure 4).

**Table 2:** The results from the automatic detected AF episodes where an ECG was stored

Category ECG inspection	Number	RMSSD Mean (SD)	Duration (minutes) Median (min‐max)
SR	12	47.5 (28.9)	6 (6‐38)
SA	28	491.6 (189.8)	8 (2‐10)
OA	14	565.3 (202.9)	2 (2‐20)
AF	46	681.5 (264.5)	12 (2‐692)

These ECGs were visually inspected and diagnosed into the following categories: Sinus rhythm (SR), Sinus arrhythmia (SA), Other arrhythmias including Atrial premature depolarisation, Ventricular premature depolarisation and Second‐degree atrio‐ventricular block (OA). Mean ± standard deviation (SD) of Root Mean Square of the Successive Differences (RMSSD) and median along with minimal and maximum values of the duration of the arrhythmia episodes registered are shown.

## 4 DISCUSSION

This study showed, that ILRs were able to diagnose PAF in Standardbred racehorses and >95% of the R waves were correctly identified by the ILRs. As we diagnosed PAF in four out of 12 horses with decreased performance, the study also indicates that PAF may occur with a higher prevalence than reported in the literature. Implantation of the ILR was fast and uncomplicated and when placed in either fifth or sixth left intercostal space, the horses were not affected by the ILR.

Paroxysmal AF has been discovered among racehorses but is sparsely described in the literature. Most PAF episodes have been diagnosed during or immediately after racing with spontaneous cardioversion within 24‐48 hours.^9^, ^10^, ^11^ Only few studies in humans have addressed the use of ILR during exercise, but although not described in detail it seems that the ILR function sufficiently.^19^, ^20^, ^21^ From this study it appears that only two PAF episodes were directly related to racing. Another three episodes occurred during race training and three episodes occurred unnoticed when the horses were at rest. We have previously shown, that the performance of ILRs in horses during exercise is reduced due to artefacts and reduced quality ECGs.^12^ In the current study, five ECGs were recorded in relation to training or racing whereas the majority were recorded at rest. The ILRs ability to detect arrhythmias during exercise warrants further investigation, but from this study it seems possible to detect AF in relation to exercise as arrhythmias were detected both before and immediately after exercise.

In people, about 5% of patients with PAF progress into persistent AF each year^22^ and further progression occurs at increasing rates with 35%‐40% of persistent AF patients developing into permanent AF within one year.^23^ The progressive nature of AF is linked to the pathophysiology of AF where each AF episode results in electrical and structural remodelling, which further increases the vulnerability of the atria to AF.^8^ It is believed that each PAF episode adds to the remodelling, eventually resulting in persistent AF.^7^ As PAF is not well‐described in horses, it is not known whether horses with persistent or permanent AF previously have had PAF episodes nor which triggering events initiated the PAF episode. In horses, APDs have been shown to act as a trigger for recurrence of AF after successful treatment,^24^ and are also involved in PAF initiation during exercise in two horses.^11^ As the ILR only records an ECG when the suspected episode of AF is already present further information about triggering events are not available.

Manual ECG inspection revealed correct identification of 46 of 100 AF episodes by the ILRs in the present study. The ILR type used in this study has an internal AF detection algorithm that operates through an assessment of the regularity of RR intervals (Lorenz plot) within a two minute interval. The Lorenz plot is graphed by plotting the difference of RR intervals against the prior difference, which in principle is similar to the Poincaré plots we used. In addition, the ILR uses an “Ectopy Rejection” which searches for exactly one P wave between two R waves.^25^ When calculating the RMSSD and plotting the Poincaré plots it was clear that the setting correctly calculated the RR intervals and based on the irregularity of these RR intervals, episodes consisting of marked SA and OAs such as VPDs were identified as AF. However, the ILRs were not always able to correctly identify the P waves in the SA and OA episodes in the equine ECGs, and therefore falsely classified them as AF events. Furthermore, the ILRs also classified 12 SR episodes as AF episodes; these were all episodes where the horses had mild tachycardia (60‐70 bpm) or where artefacts were present. This emphasises that visual inspection of the ECG traces is necessary before final AF diagnosis can be obtained with certainty in horses. In cases where false positives are evident, the Medtronic Reveal LINQ^TM^ guide suggests that the detection level is changed to a less sensitive value.^25^ By doing so, a possibility of missing true AF episodes exists and it is therefore important to decide whether there should be a high sensitivity for AF and a greater risk of false positive episodes, or a lower sensitivity for AF and a lower risk of false positive episodes. In people, the AF detecting capability possess both high sensitivity, specificity and positive predictive values when compared to Holter ECG where the recently introduced P wave evidence score in the ILRs have reduced the number of falsely detected AF episodes.^17^ In the current study, it was not possible to evaluate the sensitivity and the specificity of the ILRs as a diagnostic tool for PAF diagnosis in horses as we had no continuous ECG recording, which would serve as the gold standard. Continuous ECG recording would provide information on possible arrhythmia episodes, which the ILRs did not register and could therefore confirm or reject the arrhythmias registered by the ILR. As these were client‐owned horses and due to the intermittent occurrence of the arrhythmia over an extensive time period, Holter monitoring was not feasible.

The quality of the ECG recordings from the ILRs remained stable over time from months to years in the horses and surprisingly few ventricular beats were falsely identified, which is in agreement with human studies.^20^ Reasons for AF misdetection in people were artefacts, premature atrial or ventricular depolarisations or T wave over sensing,^13^ corresponding to our findings in horses. In horses however, 2AVB occurs frequently and this physiological rhythm also accounts for many of the misdetections seen in the horses included in this study. Surprisingly some episodes of SA were also falsely identified as AF by the ILRs. Visual inspection confirmed marked SA in these cases and the high RMSSD seen in the SA category supports the high variation in RR intervals. As only few episodes of AF were registered in the current study, manual inspection of the ECG traces was easy to perform allowing us to correct if SA or OA were falsely registered as AF.

Limitations of the study were the low number of horses included and that several episodes were registered in the same horses and included in the statistical analyses as individual variables. Whether the RMSSD is dependent on the individual horses is unknown and may therefore be a potential bias of the study. Furthermore, there was a wide difference in the amount of time every horse had the ILR implanted and also in the number of interrogations performed. Despite the irregular interrogation intervals, the episode lists were never full and except from the horse with persistent AF, all ECGs were stored and accessible for manual inspection. As we had no Holter recordings during the study period it is unknown whether the ILR missed PAF episodes and evaluation of the sensitivity was impossible. Manual inspection of the ECGs recorded by the ILRs revealed a high rate of false positive misclassifications indicative of low specificity, which is a limitation of the ILRs. However long‐term Holter recordings are required in order to evaluate this properly. Also PAF episodes of short duration (<two minutes) could be missed as the ILR only records if arrhythmia is present for two minutes or longer. Inclusion criteria of the horses could have been stricter i.e. only including horses where the poor performance occurred abruptly during exercise and where other causes had been ruled out. Some of the horses included had a history of progressive suboptimal race performance, which is not pathognomonic for previously reported PAF episodes in horses. Extended work‐up of the horses including dynamic exercise endoscopy could add insights to whether upper airway obstructions and consequently hypoxia could trigger PAF while under sympathetic influence.

## 5 CONCLUSIONS

Paroxysmal AF was diagnosed on several occasions, suggesting that ILRs can be a useful ECG tool in the clinical work‐up of horses presenting with intermittent poor performance. The frequency of PAF detected in this study (4/12 horses) was unexpectedly high despite the fact that the horses included had a history of poor performance. Whether this is true for the general horse population warrants further investigation. Whether the ILR can be used to detect PAF episodes during exercise still remains unclear as no ECGs of the episodes detected during physical activity were recorded. Despite this limitation, the method provides the opportunity for long‐term assessment of AF burden and further add to the understanding of AF development in horses. More horses, both healthy and poor performing, should be included in future studies.

## CONFLICT OF INTERESTS

No competing interests have been declared.

## AUTHOR CONTRIBUTIONS

R. Buhl and E. Hesselkilde designed the study, participated in study execution, analysed the data and drafted the manuscript. S. Nissen, M.L. Winther, S. Poulsen, C. Hopster‐Iversen and H. Carstensen participated in study execution and analysed the data. T. Jespersen and P. Sanders participated in study design and drafted the manuscript. All authors read and approved the final version of the manuscript.

## ETHICAL ANIMAL RESEARCH

The local ethical committee at the Department of Veterinary Clinical Sciences, University of Copenhagen approved the study.

## OWNER INFORMED CONSENT

Written owner‐informed consent was obtained prior to the study.

### Peer Review

The peer review history for this article is available at https://publons.com/publon/10.1111/evj.13372.

## Supporting information

Fig S1

Fig S2

Table S1

## Data Availability

The data are available from the corresponding author on reasonable request.
