# Supplementary material for: Implantable loop recorders can detect paroxysmal atrial fibrillation in Standardbred racehorses with intermittent poor performance
Source: Equine Vet J. 2020 Nov 23;53(5):955–63. doi: 10.1111/evj.13372 (PMC8451893; doi:10.1111/evj.13372)

Equine Veterinary Journal BEVA

Figure S1:

Atrial Fibrillation

A

Episode List

Device: **REVEAL LINQ LNQ11** Serial Number: **RLA400797S** Date of Visit: **01-May-2020 09:14:33**

Patient: #10 ID: **440926-3979** Physician:

Arrhythmia Episode List: 03-Mar-2020 12:47:53 to 01-May-2020 09:14:33

All collected episodes.

ID#	Type	Date	Time hh:mm	Duration hh:mm:ss	Max V. Rate	Median V. Rate
10.6	AF	24-Mar-2020	04:32	02:20:00	333 bpm (180 ms)	63 bpm (950 ms)
10.5	AF	24-Mar-2020	02:52	01:30:00	176 bpm (340 ms)	61 bpm (980 ms)
10.4	AF	24-Mar-2020	02:42	:02:00	78 bpm (770 ms)	47 bpm (1270 ms)
10.3	AF	24-Mar-2020	02:20	:16:00	167 bpm (360 ms)	61 bpm (990 ms)
10.2	AF	24-Mar-2020	01:40	:38:00	182 bpm (330 ms)	54 bpm (1120 ms)

------------------- Last Programmer Session 03-Mar-2020 -------------------

B

AF Episode #10.5

Device: **REVEAL LINQ LNQ11** Serial Number: **RLA400797S** Date of Visit: **01-May-2020 09:14:33**

Patient: #10 ID: Physician:

ID#	Type	Date	Time hh:mm	Duration hh:mm:ss	Max V. Rate	Median V. Rate
10.5	AF	24-Mar-2020	02:52	01:30:00	176 bpm (340 ms)	61 bpm (980 ms)

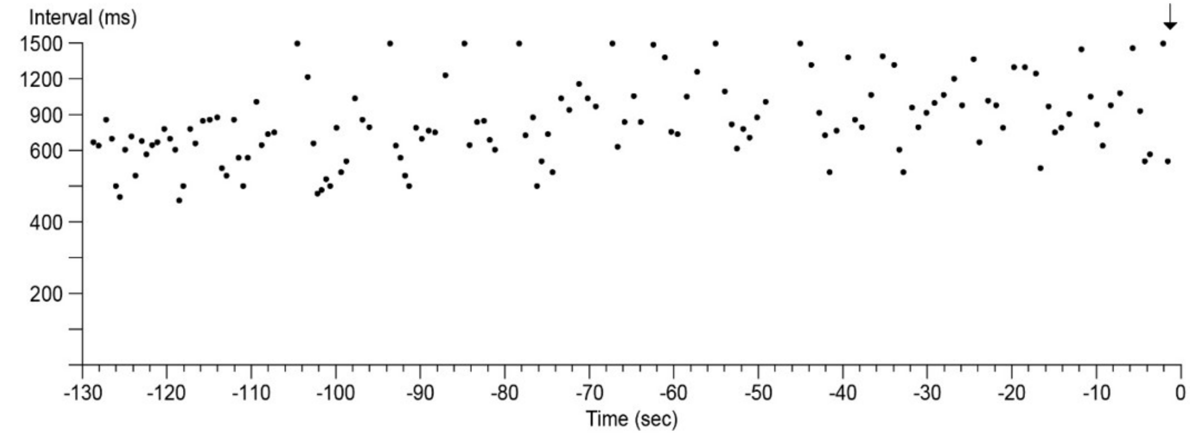

C

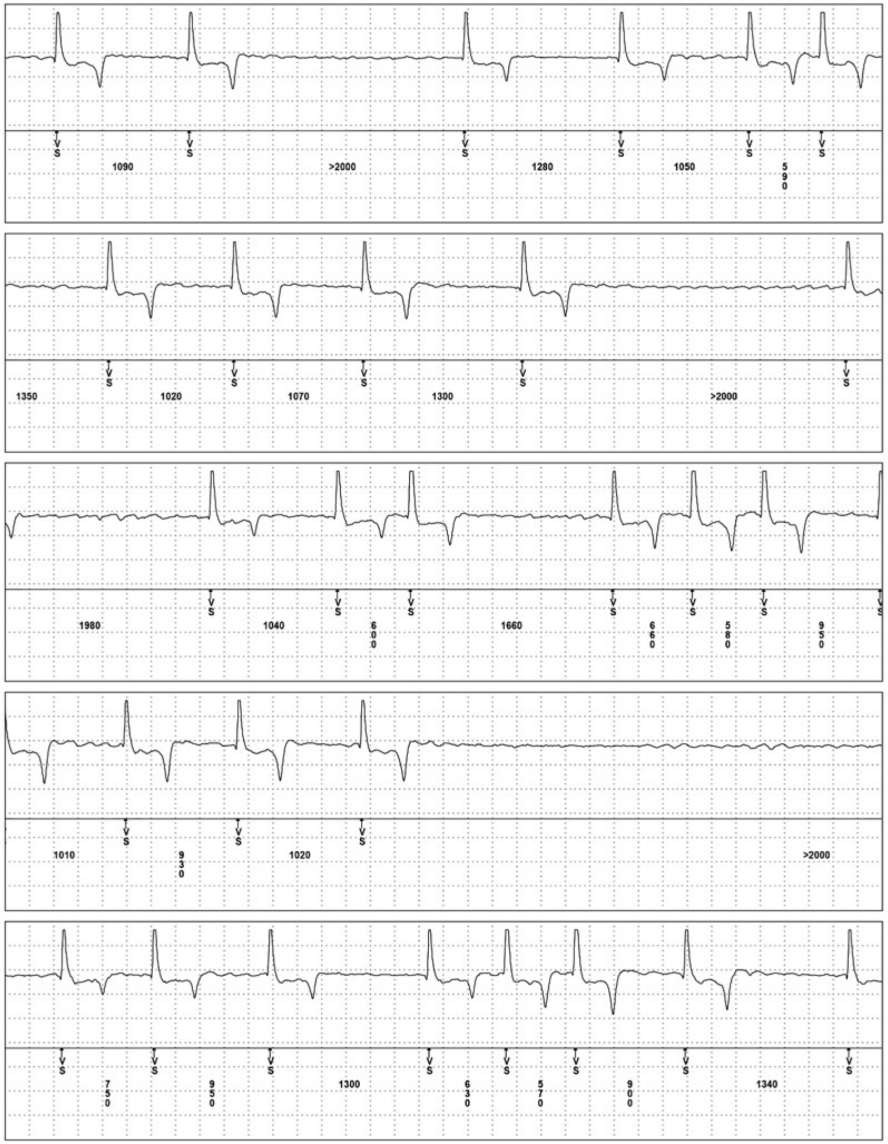

Supplement: Supplementary file 1 — Fig S1 [file EVJ-53-955-s003.pdf]
