# Supplementary material for: Implantable loop recorders can detect paroxysmal atrial fibrillation in Standardbred racehorses with intermittent poor performance
Source: Equine Vet J. 2020 Nov 23;53(5):955–63. doi: 10.1111/evj.13372 (PMC8451893; doi:10.1111/evj.13372)

Equine Veterinary Journal BEVA

Figure S2:

Ventricular Premature Complexes

A

AF Episode #11.1

Device: **REVEAL LINQ LNQ11** Serial Number: **RLA033654G** Date of Visit: **04-Mar-2020 12:33:02**

Patient: **#11** ID: Physician:

ID#	Type	Date	Time hh:mm	Duration hh:mm:ss	Max V. Rate	Median V. Rate
11.1	AF	13-Feb-2020	22:04	:02:00	167 bpm (360 ms)	79 bpm (760 ms)

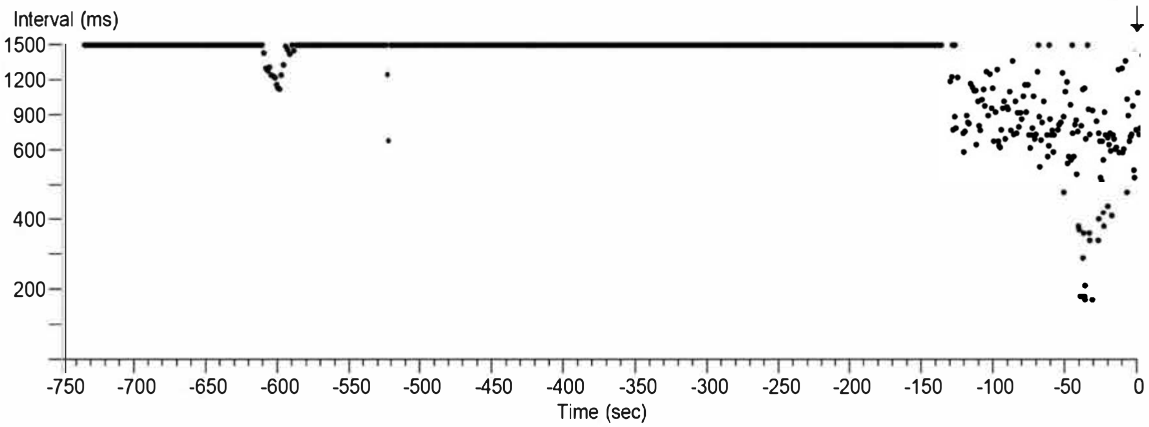

B

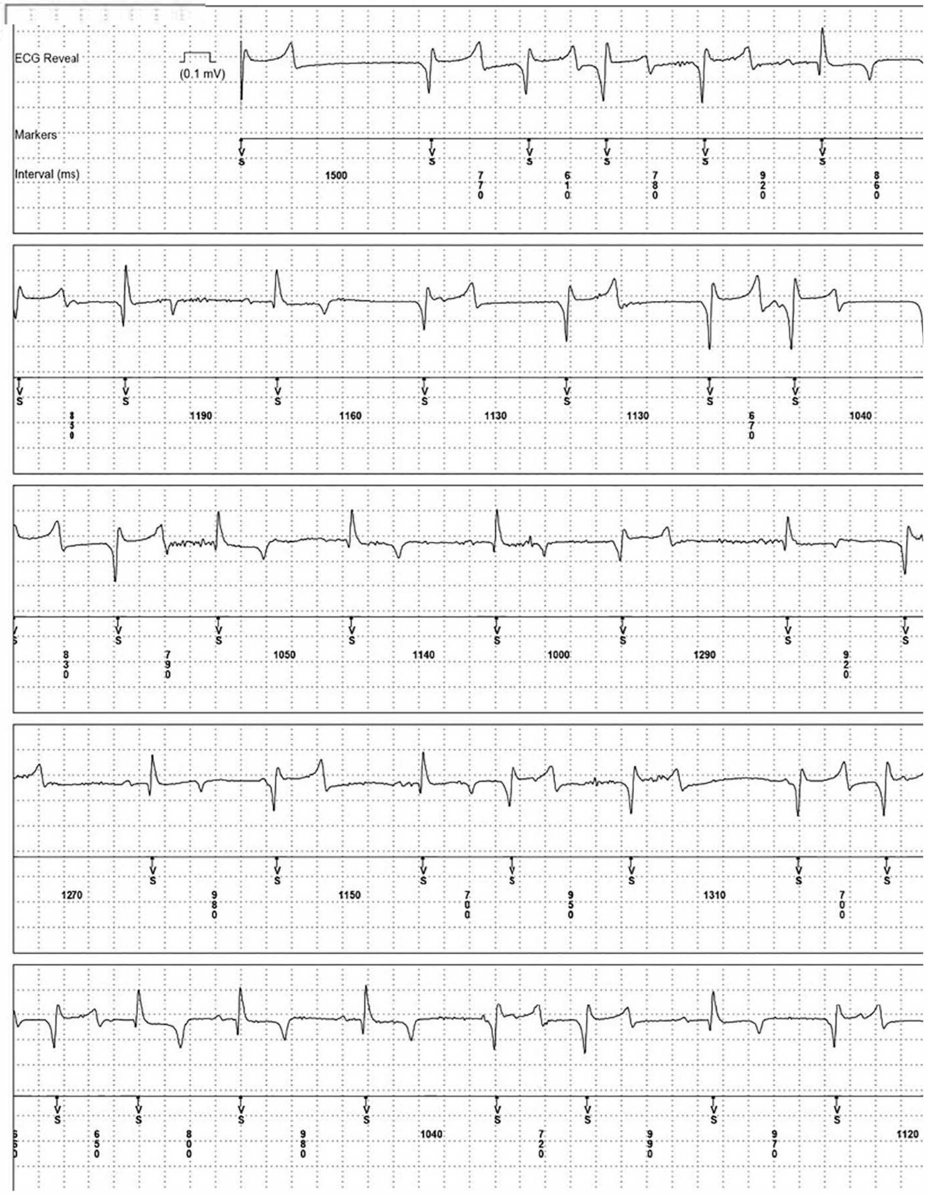

Supplement: Supplementary file 2 — Fig S2 [file EVJ-53-955-s002.pdf]
