# Supplementary material for: Implantable loop recorders can detect paroxysmal atrial fibrillation in Standardbred racehorses with intermittent poor performance
Source: Equine Vet J. 2020 Nov 23;53(5):955–63. doi: 10.1111/evj.13372 (PMC8451893; doi:10.1111/evj.13372)
Supplement: Supplementary file 3 — Table S1 [file EVJ-53-955-s001.docx]

Table S1

Classification of recorded episodes where an ECG was available as well as date, time of onset and duration of arrhythmia is shown. All episodes were categorised by the ILR as atrial fibrillation (AF). By visual inspection of the ECG traces, the arrhythmia was categorised as either sinus rhythm (SR), sinus arrhythmia (SA) or AF. In addition, other arrhythmias such as SA with second degree AV block (2AVB), atrial premature depolarisation (APD) and ventricular premature depolarization (VPD) were classified. The number of these *other arrhythmias* is annotated in brackets. For six of the SR episodes, artefacts (shown in brackets) were present resulting in oversensing of ventricular beats. *mild tachycardia were present, **moderate tachycardia were present. For each episode, the RMSSD (Root Mean Square of the Successive Differences) was calculated.

| **Horse ID** | **Episode no.** | **ILR diagnosis** | **ECG diagnosis (no. of arrhythmias)** | **RMSSD** | **Date** | **Time** | **Duration** |
| --- | --- | --- | --- | --- | --- | --- | --- |
|  |  |  |  |  |  |  |  |
| **#01**  Inclusion duration: 28 month  Number of interrogations: 10  Number of races: 32 | #01.1 | AF | SR (artefact) | 19.2 | 31.05.2018 | 04:14 | 0 h 06 min |
|  | #01.2 | AF | SR (artefact) | 96.6 | 05.06.2018 | 03.24 | 0 h 06 min |
|  | #01.3 | AF | SA | 256.0 | 21.06.2018 | 11:22 | 0 h 06 min |
|  | #01.4 | AF | AF | 1397.0 | 03.08.2018 | 05:50 | 2 h 48 min |
|  | #01.5 | AF | AF | 1045.9 | 03.08.2018 | 08:40 | 5 h 02 min |
|  | #01.6 | AF | SR*(artefact) | 14.1 | 10.10.2018 | 10:32 | 0 h 10 min |
|  | #01.7 | AF | SR | 84.6 | 16.10.2018 | 07:44 | 0 h 06 min |
|  | #01.8 | AF | AF | 194.2 | 21.12.2018 | 10:48 | 1 h 46 min |
|  | #01.9 | AF | AF | 949.7 | 21.12.2018 | 12:54 | 4 h 06 min |
|  | #01.10 | AF | AF | 1530.4 | 21.12.2018 | 17:06 | 1 h 02 min |
|  | #01.11 | AF | SR* | 42.4 | 06.01.2019 | 09:24 | 0 h 06 min |
|  | #01.12 | AF | SR* (artefact) | 30.5 | 30.01.2019 | 10:58 | 0 h 36 min |
|  | #01.13 | AF | SA* (artefact) | 137.8 | 10.06.2019 | 07.30 | 0 h 06 min |
|  | #01.14 | AF | SR* (artefact) | 80.9 | 17.09.2019 | 10:14 | 0 h 38min |
| **#02**  Inclusion duration:  7 month    Number of interrogations: 7    Number of races: 9 | #02.1 | AF | SA/2AVB (3) | 873.1 | 04.06.2019 | 06:59 | 0 h 06 min |
|  | #02.2 | AF | SA | 243.5 | 05.06.2019 | 05:25 | 0 h 06 min |
|  | #02.3 | AF | SA | 504.7 | 05.06.2019 | 04:55 | 0 h 08 min |
|  | #02.4 | AF | SR* | 43.0 | 08.07.2019 | 08:29 | 0 h 06 min |
|  | #02.5 | AF | AF | 493.7 | 21.07.2019 | 07:05 | 1 h 06 min |
|  | #02.6 | AF | AF | 748.3 | 21.07.2019 | 08:19 | 0 h 12 min |
|  | #02.7 | AF | AF | 714.2 | 21.07.2019 | 08:33 | 0 h 08 min |
|  | #02.8 | AF | AF | 674.9 | 15.10.2019 | 19:43 | 0 h 08 min |
|  | #02.9 | AF | SA | 275.5 | 10.11.2019 | 03:41 | 0 h 02 min |
|  | #02.10 | AF | AF | 315.9 | 29.11.2019 | 00:45 | 0 h 14 min |
|  | #02.11 | AF | AF | 660.1 | 29.11.2019 | 01:09 | 0 h 06 min |
|  | #02.12 | AF | AF | 805.7 | 29.11.2019 | 01:31 | 0 h 04 min |
|  | #02.13 | AF | AF | 1099.0 | 29.11.2019 | 01:47 | 0 h 02 min |
|  | #02.14 | AF | AF | 372.0 | 29.11.2019 | 02:21 | 0 h 12 min |
|  | #02.15 | AF | AF | 943.7 | 29.11.2019 | 03:19 | 0 h 02 min |
|  | #02.16 | AF | AF | 959.9 | 29.11.2019 | 05:17 | 0 h 10 min |
|  | #02.17 | AF | AF | 512.1 | 29.11.2019 | 06:35 | 0 h 02 min |
|  | #02.18 | AF | AF | 396.1 | 29.11.2019 | 07:15 | 0 h 02 min |
|  | #02.19 | AF | AF | 846.8 | 29.11.2019 | 07:23 | 0 h 04 min |
|  | #02.20 | AF | AF | 790.2 | 29.11.2019 | 07:35 | 0 h 02 min |
|  | #02.21 | AF | AF | 687.0 | 29.11.2019 | 07:51 | 0 h 02 min |
|  | #02.22 | AF | SA/2AVB (3) | 544.7 | 09.12.2019 | 04:57 | 0 h 08 min |
|  | #02.23 | AF | SA/2AVB (1) | 557.6 | 19.12.2019 | 21:31 | 0 h:22 min |
|  | #02.24 | AF | SA | 397.8 | 19.12.2019 | 22:05 | 0 h 02 min |
|  | #02.25 | AF | SA/2AVB (3) | 962.3 | 16.04.2020 | 11:23 | 0 h 02 min |
| **#03**  Inclusion duration:  12 month  Number of interrogations: 7    Number of races: 12 | #03.1 | AF | SR** (artefact) | 23.0 | 16.11.2019 | 12:16 | 0 h 10 min |
|  | #03.2 | AF | SR** | 29.8 | 24.12.2019 | 11:16 | 0 h 06 min |
|  | #03.3 | AF | SA | 431.9 | 29.02.2020 | 21:40 | 0 h 02 min |
|  | #03.4 | AF | SA | 340.0 | 01.03.2020 | 04:36 | 0 h 08 min |
|  | #03.5 | AF | VPC (55) | 512.2 | 01.03.2020 | 10:59 | 0 h 04 min |
|  | #03.6 | AF | VPC (55) | 605.7 | 01.03.2020 | 12:48 | 0 h 02 min |
|  | #03.7 | AF | VPC (45) | 583.8 | 01.03.2020 | 14:24 | 0 h 04 min |
|  | #03.8 | AF | VPC (63) | 627.8 | 01.03.2020 | 17:12 | 0 h 02 min |
|  | #03.9 | AF | VPC (74) | 457.2 | 01.03.2020 | 17:18 | 0 h 02 min |
|  | #03.10 | AF | VPC (73) | 443.9 | 01.03.2020 | 22:46 | 0 h 02 min |
|  | #03.11 | AF | VPC (56) | 477.4 | 01.03.2020 | 22:52 | 0 h 02 min |
|  | #03.12 | AF | SR** | 75.8 | 01.03.2020 | 16:00 | 0 h 08 min |
|  | #03.13 | AF | SR* (artefact) | 29.5 | 28.04.2020 | 16:08 | 0 h 32 min |
| **#04**  Inclusion duration:  12 month    Number of interrogations: 7    Number of races: 19 | No episodes |  |  |  |  |  |  |
| **#05**  Inclusion duration:  9 month    Number of interrogations: 7    Number of races: 9 | #05.1 | AF | AF | 581.8 | 20.08.2019 | 15:30 | 7 h 24 min |
|  | #05.2 | AF | AF | 804.3 | 26.08.2019 | 01:58 | 0 h 08 min |
|  | #05.3 | AF | AF | 986.0 | 26.08.2019 | 02:08 | 1 h 26 min |
|  | #05.4 | AF | AF | 963.4 | 26.08.2019 | 04:24 | 1 h 28 min |
|  | #05.5 | AF | AF | 475.6 | 26.08.2019 | 06:20 | 0 h 14 min |
|  | #05.6 | AF | AF | 603.2 | 26.08.2019 | 07:14 | 0 h 06 min |
|  | #05.7 | AF | AF | 791.2 | 26.08.2019 | 07:32 | 0 h 08 min |
|  | #05.8 | AF | AF | 765.5 | 26.08.2019 | 07:52 | 0 h 06 min |
|  | #05.9 | AF | AF | 749.1 | 26.08.2019 | 08:18 | 0 h 06 min |
|  | #05.10 | AF | AF | 887.4 | 26.08.2019 | 09:06 | 0 h 08 min |
|  | #05.11 | AF | AF | 723.7 | 26.08.2019 | 11:36 | 0 h 08 min |
|  | #05.12 | AF | AF | 775.5 | 26.08.2019 | 12:02 | 0 h 08 min |
|  | #05.13 | AF | AF | 675.1 | 26.08.2019 | 12:34 | 0 h 22 min |
|  | #05.14 | AF | AF | 646.0 | 26.08.2019 | 13:24 | 0 h 06 min |
|  | #05.15 | AF | AF | 845.8 | 27.08.2019 | 00:34 | 1 h 56 min |
|  | #05.16 | AF | AF | 729.9 | 27.08.2019 | 04:18 | 1 h 28 min |
|  | #05.17 | AF | AF | 615.3 | 27.08.2019 | 13:28 | 4 h 34 min |
|  | #05.18 | AF | SA | 691.2 | 07.09.2019 | 03:36 | 0 h 20 min |
|  | #05.19 | AF | SA | 629.8 | 08.09.2019 | 06:46 | 0 h 12 min |
|  | #05.20 | AF | SA | 748.5 | 08.09.2019 | 08:02 | 0 h 06 min |
|  | #05.21 | AF | SA | 725.2 | 08.09.2019 | 11:10 | 0 h 06 min |
|  | #05.22 | AF | SA | 682.1 | 08.09.2019 | 14:02 | 0 h 08 min |
|  | #05.23 | AF | SA | 672.4 | 08.09.2019 | 16.04 | 0 h 06 min |
|  | #05.24 | AF | SA | 610.3 | 08.09.2019 | 17:06 | 0 h 10 min |
|  | #05.25 | AF | SA | 562.6 | 08.09.2019 | 22:20 | 0 h 08 min |
|  | #05.26 | AF | SA | 674.4 | 08.09.2019 | 22:46 | 0 h 06 min |
|  | #05.27 | AF | SA | 742.6 | 09.09.2019 | 03:56 | 0 h 08 min |
|  | #05.28 | AF | SA | 613.5 | 09.09.2019 | 04:46 | 0 h 08 min |
|  | #05.29 | AF | SA | 380.7 | 10.09.2019 | 04:54 | 0 h 10 min |
|  | #05.30 | AF | SA | 633.5 | 12.09.2019 | 04:46 | 0 h 12 min |
|  | #05.31 | AF | SA | 376.9 | 24.09.2019 | 01:48 | 0 h 08 min |
|  | #05.32 | AF | SA | 324.2 | 13.10.2019 | 01:54 | 0 h 06 min |
|  | #05.33 | AF | SA | 471.2 | 18.10.2019 | 00:20 | 0 h 08min |
|  | #05.34 | AF | SA | 570.1 | 20.10.2019 | 04:34 | 0 h 10 min |
|  | #05.35 | AF | SA | 480.9 | 22.10.2019 | 05:08 | 0 h 06 min |
|  | #05.36 | AF | SA | 527.8 | 10.11.2019 | 07:14 | 0 h 10 min |
| **#06**  Inclusion duration:  7 month    Number of interrogations: 4    Number of races: 9 | #06.1 | AF | SA | 58.8 | 20.12.2019 | 14:02 | 0 h 04min |
| **#07**  Inclusion duration:  7 month    Number of interrogations: 5    Number of races: 12 | No episodes |  |  |  |  |  |  |
| **#08**  Inclusion duration:  6 month    Number of interrogations: 3    Number of races: 1 | No episodes |  |  |  |  |  |  |
| **#09**  Inclusion duration:  6 month    Number of interrogations: 3    Number of races: 3 | No episodes |  |  |  |  |  |  |
| **#10**  Inclusion duration:  21 month    Number of interrogations: 3    Number of races:30 | #10.1 | AF | SA/APC (1) | 192.2 | 27.08.2019 | 12:36 | 0 h 02 min |
|  | #10.2 | AF | AF | 636.6 | 24.03.2020 | 01:40 | 0 h 38 min |
|  | #10.3 | AF | AF | 835.7 | 24.03.2020 | 02:20 | 0 h 16 min |
|  | #10.4 | AF | AF | 898.1 | 24.03.2020 | 02:42 | 0 h 02 min |
|  | #10.5 | AF | AF | 652.0 | 24.03.2020 | 02:52 | 1 h 30 min |
|  | #10.6 | AF | AF | 601.0 | 24.03.2020 | 04:32 | 2 h 30 min |
| **#11**  Inclusion duration:  7 month    Number of interrogations: 3    Number of races: 9 | #11.1 | AF | VPC (97) | 315.7 | 13.02.2020 | 22.04 | 0 h 2 min |
| **#12**  Inclusion duration:  8 month    Number of interrogations: 3    Number of races: 13 | 0,56 | AF | AF | 631.9 | 09.12.2019 | 19:17 | 2 h 38 min |
|  | #12.2 | AF | AF | 150.6 | 09.12.2019 | 21:57 | 7 h 18 min |
|  | #12.3 | AF | VPC/2AVB (90)/(2) | 760.1 | 15.12.2019 | 15:47 | 0 h 02 min |
|  | #12.4 | AF | AF | 172.8 | 28.04.2020 | 16:21 | 11 h 32 min |
